# Does intensive home treatment change treatment trajectories of psychiatric disorders?

**DOI:** 10.1192/j.eurpsy.2023.406

**Published:** 2023-07-19

**Authors:** A. Martín-Blanco, A. González-Fernández, A. Farré, S. Vieira, P. Alvaro, C. Isern, D. Giménez, C. Torres, V. de la Cruz, C. Martín, N. Moll, O. Castro, M. Sagué-Vilavella

**Affiliations:** 1 UHPAD, Hospital de la Santa Creu i Sant Pau; 2 Grup de Recerca en Salut Mental, IIB SANT PAU, Barcelona; 3 CIBERSAM, ISCIII, Madrid; 4 UHPAD, CPB - Serveis Salut Mental; 5 Psychiatry and Psychology, Hospital Clínic de Barcelona, Barcelona, Spain

## Abstract

**Introduction:**

Intensive home treatment (IHT) for people experiencing a mental health crisis has been progressively established in many western countries as an alternative to in-ward admission. But is this a real alternative? We previously reported that patients treated in our IHT unit only differ from those voluntarily admitted to hospital in suicidal risk and severe behaviour disorders (not in other factors such as clinical severity) (Martín-Blanco *et al.*, Rev Psiquiatr Salud Ment 2022;15:213-5). Now we are interested in disentangle if those patients who used to require inward management can be successfully treated at home.

**Objectives:**

To describe subsequent treatment trajectories of the first 1000 admissions to our IHT unit and to compare clinical characteristics among the different groups of trajectories.

**Methods:**

Retrospective cohort study. Subsequent treatment trajectories were collected from December 2016 to October 2022 and classified: absence, hospital, IHT, and mixed (hospital and IHT). Statistical significance was tested by means of ANOVA or Kruskal-Wallis test for quantitative variables (corrected for multiple comparisons) and chi-square tests for qualitative variables.

**Results:**

Tables 1 shows the characteristics of the whole sample. Of the 1000 IHT admissions, 12.1% needed subsequent hospital admission(s), 12.7% IHT admission(s), and 9.3% mixed admission(s). There were no differences among these groups in median severity at IHT admission, but there were differences in the number of previous admissions (p=0.0001): the group with no subsequent admissions had less previous admissions than the other groups (pBonf<0.0001), and the group with subsequent IHT admissions had less than the group with mixed admissions (pBonf=0.0123). There were differences between groups regarding distribution of diagnoses (p<0.0001) (Fig. 1). When considering subsequent admissions by diagnosis, there were differences in severity at IHT admission (p=0.0068) and in number of previous hospitalizations (p<0.0001) (Fig. 2).

**Table 1.** Clinical characteristics of the whole sample (N=1000)

	mean	SD
Age (years)	47.07	17.02
CGI-s at admission *	5	4-5
	N	%
Sex (female)	548	54.8%
Psychotic disorders	463	46.3%
Affective disorder	257	25.7%
Bipolar disorder	128	12.8%
Other disorders	152	15.2%
Hospital admission in the previous 5 years	313	31.3%

CGI-s: clinical global impression - severity. * median and IQR

**Image:**

**Figure:**
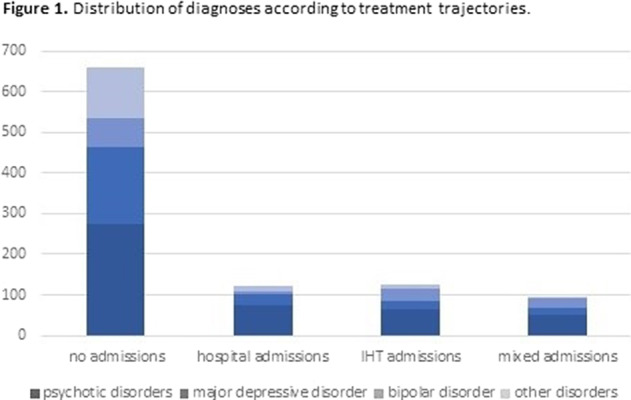

**Image 2:**

**Figure:**
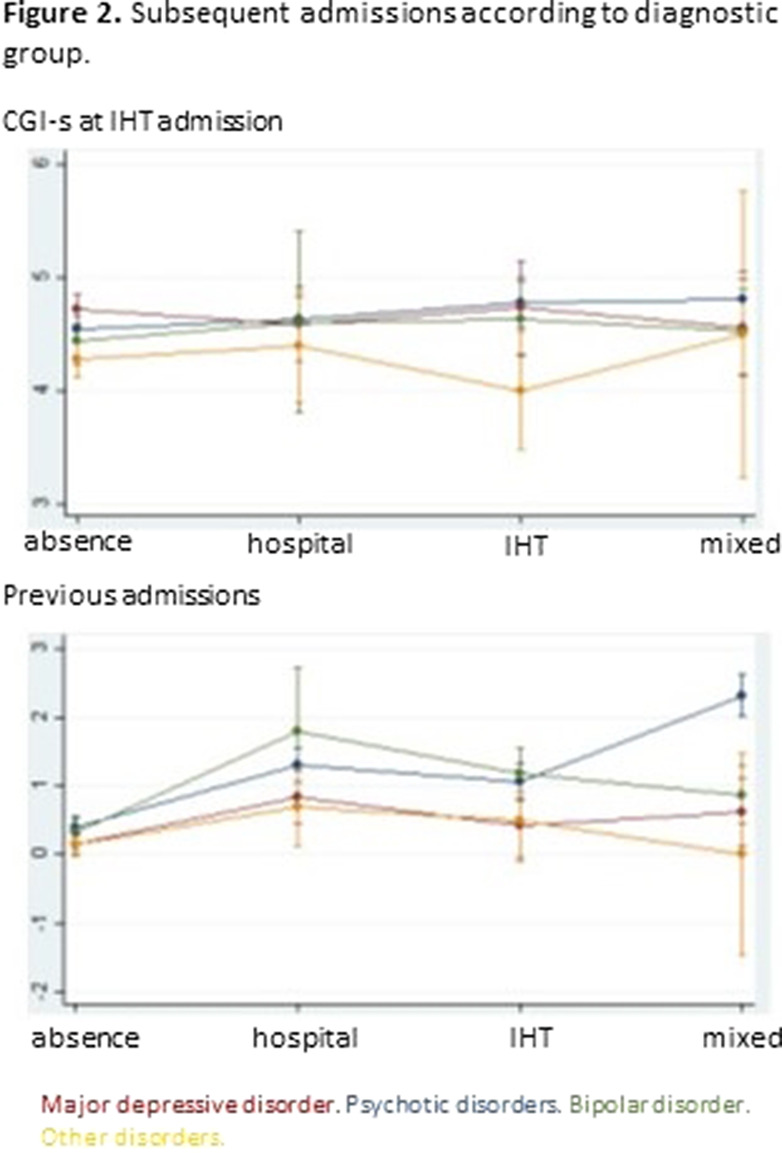

**Conclusions:**

Patients that used to require inward management can now be treated at home when suffering an acute episode. Therefore, IHT has changed treatment trajectories for some patients with psychiatric disorders.

**Disclosure of Interest:**

None Declared

